# Association of miR-146a and miR196a2 genotype with susceptibility to idiopathic recurrent pregnancy loss in Iranian women: A case-control study

**DOI:** 10.18502/ijrm.v19i8.9620

**Published:** 2021-09-09

**Authors:** Emad Babakhanzadeh, Hamid Danaei, Mohammad Abedinzadeh, Hamid Reza Ashrafzadeh, Nasrin Ghasemi

**Affiliations:** ^1^Department of Medical Genetics, Shahid Sadoughi University of Medical Sciences, Yazd, Iran.; ^2^Medical Genetics Research Center, Shahid Sadoughi University of Medical Sciences, Yazd, Iran.; ^3^Abortion Research Centre, Yazd Reproductive Sciences Institute, Shahid Sadoughi University of Medical Sciences, Yazd, Iran.

**Keywords:** RPL, miR-146a, miR196a2, Polymorphism, RFLP.

## Abstract

**Background:**

Recurrent pregnancy loss (RPL) is the most common complaint of pregnancy in females with a prevalence of 5%. Numerous documents have shown that single nucleotide polymorphisms are able to change miRNA transcription and/or maturation, which may alter the incidence of disorders such as RPL.

**Objective:**

To assess the relationship of miR-146aC > G (rs2910164) and miR-196a2T > C (rs11614913) with RPL susceptibility in Iranian women.

**Materials and Methods:**

Blood samples were collected from 214 women who had experienced at least two consecutive spontaneous miscarriages (case) and 147 normal individuals without a history of miscarriage (control). MiR-146aC > G and miR-196a2T > C genotypes were evaluated via the restriction fragment length polymorphism technique.

**Results:**

The genotypes incidence did not show a significant difference in pre-miR-146aC > G polymorphism CC vs CG + GG (p = 0.854; OR = 0.933; 95% CI) and CC + CG vs GG (p = 0.282; OR = 1.454; 95% CI). Also, no significant difference was observed between pre-miR-196a2T > C polymorphism TT vs TC + CC (p = 0.862; OR = 0.938; 95% CI) and TT + TC vs CC and (p = 0.291; OR = 1.462; 95% CI) in both the case and control groups.

**Conclusion:**

The results showed that although the distribution of miR-146aC > G and miR-196a2T > C was different between the unknown RPL and control groups, these variances were not statistically significant.

## 1. Introduction

Recurrent pregnancy loss (RPL), with a prevalence of 5% in women, is the most common pregnancy complication and significantly affects the quality of human life (1-4). Researchers have focused on the causes of recurrent miscarriage and the efforts to resolve this problem (5, 6). Various factors such as infection, chromosomal abnormalities, immune disorders, and endocrine and anatomical dysfunction are involved in RPL (6, 7). Despite several known factors influencing RPL, there is still no obvious cause, which psychologically affects many couples (8-10).

MicroRNAs are fragments of small, single-stranded, non-coding RNAs 22-26 nucleotides in length that are involved in post-transcriptional control (11-14). Many studies have confirmed the association of miRNAs with endometriosis, ovarian dysfunction, and infertility. For instance, mir-21, mir-24, and mir-26 play an important role in the occurrence of these phenomena (15, 16). Numerous documents have shown that single nucleotide polymorphisms (SNPs) can alter miRNA transcription and/or maturation, which may then alter the incidence of disorders (17-19).

Mir-125a is the negative regulator of the LIF Receptor Subunit Alpha *(LIFR)* and Erb-B2 Receptor Tyrosine Kinase 2 (*ERBB2*) genes, which play a critical roles in embryo implantation (20). In was observed in a study that two SNPs in pri-miR-125a (rs41275794 and rs12976445) were involved in RPL in the Han-Chinese population (18). An experiment examined the relationship of SNPs in miR-146a (rs2910164) and miR-196 (rs11614913) with the occurrence of RPL in Korean women and found that miR-196a2T > C had a positive correlation with RPL (17). *Fas* cell surface death receptor *(FAS*) (21) and Homeobox B8 *(HOXB8)* (22) are the target genes of miR-146a and miR-196, respectively, and are involved in apoptosis and inhibition of myeloid differentiation (23).

The purpose of this study was to assess the relationship of miR-146aC > G (rs2910164) and miR-196a2T > C (rs11614913) with RPL susceptibility in Iranian women due to the influence of genetic factors on spontaneous abortion, as well as the association of miR-146aC > G (rs2910164) and miR-196a2T > C (rs11614913) with RPL sensitivity in different ethnic groups.

## 2. Materials and Methods

### Study subjects

In this case-control study, peripheral blood samples (5 ml) were collected from 214 women with idiopathic RPL and a history of at least two sequential spontaneous miscarriages (mean age: 31.6 ± 3.42 yr; age range: 21-43 yr, and body mass index: 20.8 ± 2.16 kg/m${}^{2}$) and 147 controls with no history of miscarriage (mean age: 31.2 ± 6.34 yr; age range: 21-42 yr, and body mass index: 20.8 ± 2.85 kg/m${}^{2}$). All participants were referred to the Abortion Research Centre, Yazd Reproductive Sciences Institute, Shahid Sadoughi University of Medical Sciences, Yazd, Iran between November 2018 and April 2019. Women with RPL due to chromosomal abnormalities (in the patients or their spouses), immune disorders, or endocrine or anatomical dysfunction were excluded from the study. Some samples were also excluded due to the poor quality of the extracted DNA.

### Genotyping

To obtain the genomic DNA, the Qiagen kit (QIAamp DNA Blood Mini Kit; Cat No: 51105) was used. The quality and quantity of extracted DNA were determined by agarose gel and spectrophotometer (OD 260/280), respectively. Determination of the MiR-146aC > G and miR-196a2T > C genotypes was performed by the polymerase chain reaction (PCR)-restriction fragment length polymorphism technique. PCR of the miR-146aC > G and miR-196a2T > C genotypes was performed using the following primers: forward 5'- CATGGGTTG TGTCAG TGTCAGAGCT-3' and reverse 5'-TGC CTA CTG TCA CCA GTC TTC CAA-3' for the miR-149T > C genotype, and forward 5'-CTGGCTCCGTGTCTTCACTC-3' and reverse 5'-TGAGGCCCG AAACACCCG TA-3' for the miR-196a2T > C genotype. After amplification, the PCR products were digested by restriction endonucleases *SacI* and *MspI*, respectively; 3% agarose gel and horizontal electrophoresis were used to detect the enzymatic digestion and this was visualized immediately in ultraviolet illumination to investigate the genotype.

In the enzymatic digestion of the PCR product for miR-146a by *SacI*, we expected to find three bands in the heterozygous samples (GC genotype) with lengths of 147, 125, and 22 bp.

**Figure 1:**
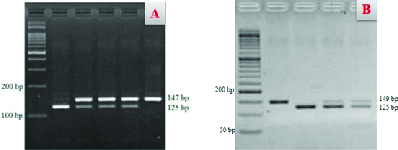
PCR product results after enzymatic digestion in 3% agarose. (A) Enzymatic digestion of PCR product for miR-146a by *SacI*. (B) Enzymatic digestion of PCR product for miR-196a2 by *MspI*.

As presented in Figure 1A, while the 147- and 125-bp bands were detected, the 22-bp band was not detectede in agarose gel due to its smaller length. In the mutant homozygous samples (GG genotype), we expected to find two bands with lengths of 125 and 22-bp. As shown in Figure 1A, the 125-bp band was visible, while the 22-bp band was not detected because of its small length. In the wild-type homozygous samples (CC), a sharp band (147 bp) was detected. For the enzymatic digestion of the PCR product for miR-196a2 by *MspI*, we anticipated to find three bands in the heterozygous samples (CT genotype) with lengths of 149, 125, and 24 bp. As presented in Figure 1B, the 149- and 125-bp bands were visible, but the 24-bp band was not detected in agarose gel due to its smaller length. In the mutant homozygous samples (CC genotype), we expected to find two bands with lengths of 125 and 24 bp. As shown in Figure 1A, while the 125-bp band was visible, the 24-bp band was not detected because of its small length. In the wild-type homozygous samples (TT), a sharp band (149bp) was detected.

### Ethical considerations

This study was approved by the Institutional Ethics Committee of the Ashkezar Azad University of Yazd, Yazd, Iran. A written informed consent was obtained from each participant before the collection of tissue samples.

### Statistical analysis

Statistical analysis was done using the statistical package for the social sciences (SPSS) statistical software (Inc., Chicago, IL, version 18). To assess the variances of allele and genotype frequencies, the Chi-square test was used. Logistic regression was used to calculate the odds ratio (OR) and 95% confidence intervals (CI). P < 0.05 was considered as statistically significant.

## 3. Results 

Table I presents the miR-146aC > G and miR-196a2T > C distributions in the RPL and control groups. Although the distributions of the miR-146aC > G polymorphism were different between the unknown RPL and control groups, the difference was not statistically significant. The highest and lowest frequency alleles observed in both groups were found by determining the allele frequency as C (F = 73%) and G (F = 27%) in the control group, respectively.

The results of the miR-196a2T > C genotype were similar to the miR-146aC > G polymorphism, and despite the differences in groups, no significant difference was observed. The highest and lowest allele frequencies observed in both groups were T (F = 72%) and C (F = 28%) alleles in the control group, respectively (Table II).

**Table 1:** The genotype distribution of miR-146aC > G and miR-196a2T > C polymorphisms between groups


**Feature**	**Case (n = 214)**	**Controls (n = 147)**	**OR (95% CI)**	**p-value**
**miR-146aC > G**				
**CC**	92 (43)	78 (53)	1.000 (reference)	-
**CG**	105 (49)	59 (40)	1.477	0.128
**GG**	17 (8)	10 (7)	0.089	0.863
**Dominant (CC vs CG + GG)**	- -	0.933	0.854
**Recessive (CC + CG vs GG)**	- -	1.454	0.282
**miR-196a2T > C**				
**TT**	11 (5)	15 (10)	1.000 (reference)	-
**TC**	99 (46)	70 (47)	0.953	0.850
**CC**	104 (49)	62 (43)	0.487	0.138
**Dominant (TT vs TC + CC)**	- -	0.938	0.862
**Recessive (TT + TC vs CC)**	- -	1.462	0.291
Data presented as n (%). Chi-square test, p-value < 0.01 considered as statistical significance				

**Table 2:** The allele frequencies of miR-146aC > G and miR-196a2T > C polymorphisms between groups with Chi-square test


**Feature**	**Case (n = 428)**	**Controls (n = 294)**	**OR (95% CI)**	**p-value**
**miR-146aC > G**				
**C**	291 (68)	214 (73)	1.000 (reference)	-
**G**	137 (32)	80 (27)	1.236	0.190
**miR-196a2T > C**				
**T**	282 (66)	212 (72)	1.000 (reference)	-
**C**	146 (34)	82 (28)	0.772	0.179
Data presented as n (%). Chi-square test, p-value < 0.01 considered as statistical significance				

## 4. Discussion

The current study aimed to evaluate the possible roles of pre-miR-146aC > G and pre-miR-196a2T > C polymorphisms in the etiology of unknown RPL. The results of this study did not detect a significant difference in pre-miR-146aC > G polymorphisms between the case and control individuals. Similar to our result, in the experiment of 461 Korean cases and 447 controls and in the study of 300 North Indian cases and 200 controls, it was observed that no difference in pre-miR-146aC > G SNPs between the RPL and control groups (17, 24). Numerous studies have shown that miR-146a regulates *FAS* by binding to the FAS region of mRNA 3´- UTR (21, 25).

MiR-146a transcription is controlled by the NF-kB signaling pathway, and high levels of mir-146a increase the stability of mesenchymal stem cells (21). SMAD4 is the other main target of mir-146a, which acts to maintain the undifferentiated condition of human embryonic stem cells by controlling the p21/WAF1/Cip1 promoter (26, 27).

The findings of our study also did not show a significant difference in the pre-miR-196a2T > C polymorphisms between the case and control groups. Similarly, it was found that there was no significant difference in pre-miR-196a2T > C polymorphisms between the RPL and control groups in Tabriz women (28). However, contrary to the findings of the present study, a study showed significant significantly different cases of miR-196a2 CC in the RPL vs. control group (17).

The relationship between miRNA polymorphisms and RPL has been stated in a few studies (29, 30). Pre-miRNA SNPs (miR-146aC > G and miR-196a2T > C) have been shown to be strongly associated with different kinds of cancer (31).

This suggests that these polymorphisms might have a major effect on RPL through cell proliferation, as the main cause of cancer is improper control of the cell cycle (32, 33). The condition of the endometrium in women who experience RPL can change due to abnormal release. Irregular cell proliferation in RPL, including increased hypersensitivity to abortions related to gene polymorphisms involved in cell cycle control, has been reported in some studies (34, 35).

In addition, an experiment that evaluated the potential risk of miR-196a2 SNPs between RPL and control individuals in Indian women, it was revealed an increased frequency of miR-196a2 T > C in patients (24). Previous reports showed an important function of miRNA in RPL cell cycle progression (36, 37). MiR-196a2 is associated with abortion through transcription of the HOX gene family and Akt-signaling pathway (38). HOXB8, which targets miR-196a2, participates in the myeloid differentiation and implantation process (39).

## 5. Conclusion

Considering the aforementioned points, the miR-146a and miR-196a2 targets are associated with cell proliferation and development. Although the distribution of miR-146aC > G and miR-196a2T > C were different between the unknown RPL and control groups, these difference were not statistically significant. The results of the present study are similar to the results of previous studies but differ in some respects.

This was probably due to the differences in the ethnicity of the study groups. However, more research is necessary to elucidate the positive or negative relationships between these polymorphisms and RPL in women of varied ethnicities.

##  Conflict of Interest

All authors declare that they have no financial interest with respect to this study.
